# Characterization of a New Transposon, Tn*6696*, on a *bla*_NDM–__1_-Carrying Plasmid From Multidrug-Resistant *Enterobacter cloacae* ssp. *dissolvens* in China

**DOI:** 10.3389/fmicb.2020.525479

**Published:** 2020-09-15

**Authors:** Qichao Chen, Yanfeng Lin, Zhonghong Li, Lanfen Lu, Peihan Li, Kaiying Wang, Lang Yang, Hui Ma, Peng Li, Hongbin Song

**Affiliations:** ^1^Center for Disease Control and Prevention of People’s Liberation Army, Beijing, China; ^2^Academy of Military Medical Sciences, Beijing, China; ^3^College of Environmental and Chemical Engineering, Nanchang Hangkong University, Nanchang, China; ^4^Department of Laboratory Diagnosis, Sun Yat-sen University Affiliated Zhongshan Hospital, Zhongshan, China; ^5^The Sixth Medical Center of People’s Liberation Army General Hospital, Beijing, China

**Keywords:** *Enterobacter cloacae*, carbapenem resistance, *bla*_NDM__1_, Tn*6696*, whole genome sequencing

## Abstract

**Background:**

*Enterobacter cloacae* is an opportunistic pathogen which is responsible for serious nosocomial infections. A gene which plays an important role in resistance to carbapenems is the New Delhi metallo-β-lactamase 1 (NDM-1). Currently, the spread of NDM-1-producing *E. cloacae* strains is a serious public threat.

**Methods:**

A multidrug-resistant *E. cloacae* ssp. *dissolvens* strain CBG15936 was recovered in 2017 in Guangzhou, China. PCR, S1-pulsed-field gel electrophoresis, and Southern blotting were performed to locate the *bla*_NDM__–__1_ gene. Susceptibility testing and conjugation experiments were also performed. Illumina HiSeq and Nanopore sequencers were used to perform whole-genome sequencing.

**Results:**

Strain CBG15936 belongs to ST932 and is resistant to carbapenems. The *bla*_NDM–__1_ gene was found on a ∼62-kb plasmid, which has a conjugation frequency of 1.68 × 10^–3^ events per donor cell. Genome sequencing and analysis revealed that the NDM-1-carrying IncN1 plasmid contained a new transposon Tn*6696*, which consists of an intact *qnrS1*-carrying Tn*6292* element, an inverted 8.3-kb Tn*3000* remnant, IS*kpn19*, Δ*tnpA*, and IS*26*.

**Conclusion:**

A new transposon, Tn*6696*, has been detected on a *bla*_NDM–__1_-carrying plasmid recovered from multidrug-resistant *E. cloacae* ssp. *dissolvens* CBG15936 from China. This finding provides a new perspective regarding the potential for *bla*_NDM–__1_ to undergo horizontal transfer among drug-resistant bacteria.

## Introduction

*Enterobacter cloacae* is a gram-negative opportunistic pathogen which belongs to *Enterobacteriaceae*. Previous studies have reported that *E. cloacae* are ubiquitous in nature and can be isolated from clinical samples (Davin-Regli et al., 2019). With the extensive use of antibiotics, carbapenem-resistant *E. cloacae* have become an important nosocomial pathogen which can cause septicemia and lower respiratory tract infections (Mayhall et al., 1979; Daw et al., 1989). In recent years, carbapenem-resistant *E. cloacae* isolates have been reported worldwide (Davin-Regli and Pagès, 2015). New Delhi metallo-β-lactamase 1 (NDM-1) was first detected in a Swedish patient transferred from India, and it plays a major role in the appearance and dissemination of carbapenem-resistant gram-negative bacteria (Yong et al., 2009; Jamal et al., 2013). NDM-1 has become a burden on the health care system especially in intensive care units (Ahmad et al., 2018; Khalid et al., 2019). The detection rate of NDM-1 in *E. cloacae* has been increasing globally (Karlowsky et al., 2017). Prevalence of NDM-1-carrying *E. cloacae* was observed in France (Perry et al., 2011) and Mexico (Torres-Gonzalez et al., 2015). Genome sequencing and analysis also revealed an NDM-1 *E. cloacae* outbreak in a hospital in the UK (Fairley et al., 2019). In the southwest region of China, 132 carbapenem-resistant *E. cloacae* isolates were obtained from patients between 2012 and 2016. Twenty (15.2%) of these strains were identified as NDM-1 positive (Jia et al., 2018). It is important to monitor the spread of *bla*_NDM–__1_ among *E. cloacae* stains.

Transposons are mobile genetic elements which are able to translocate chromosome or plasmids. Transposons have been shown to carry drug resistance genes and provide antibiotic resistance in pathogenic bacteria (Whittle et al., 2002). Tn*6292* is a Tn-family unit transposon which belongs to the Tn3-family and has an IS*26* at the right end. In addition, Tn*6292* contains a quinolone resistance region *qnrS1* (Feng et al., 2016). Multidrug-resistant (MDR) bacteria containing Tn*6292* have been reported many times in China (Li et al., 2018). Tn*3000* is bracketed by IS*3000* at both ends and contains the *bla*_NDM–__1_ gene. Tn*3000* has been shown to be responsible for transmission of the *bla*_NDM–__1_ gene in various parts of the world (Campos et al., 2015). Thus, it is important to elucidate the genetic features of transposons in order to explore possible mechanisms of bacterial resistance.

Therefore, in this study, we report a carbapenem-resistant *E. cloacae* ssp. *dissolvens* strain and the genetic features of the *bla*_NDM–__1_-harboring plasmid it carries. In addition, we identify a new transposon, Tn*6696*, present in this plasmid. These findings provide a new perspective regarding possible mechanisms of gene transmission to mediate drug resistance.

## Materials and Methods

### Bacterial Identification

Strain CBG15936 was recovered from the sputum of a patient in Guangzhou, China, in 2017. The strain was identified by using the Vitek 2 Compact System (BioMérieux, France) and confirmed with 16S rRNA gene sequencing. The *bla*_NDM–__1_ gene was detected by PCR amplification as previously described (Zhang et al., 2013). This isolate was collected through routine surveillance, and verbal consent was obtained as no personally identifiable data were included. The ethics of the study was reviewed and supervised by the Center for Disease Control and Prevention of PLA. All experiments were performed in the biosafety cabinet following the standard procedure.

### S1-Pulsed-Field Gel Electrophoresis (PFGE) and Conjugation Experiments

Genomic DNA was prepared in agarose plugs and digested with the S1 endonuclease (Takara, Dalian, China). DNA fragments were electrophoresed on a CHEF-DR III system (Bio-Rad, Hercules, United States) for 15 h at 14°C with run conditions of 6 V/cm and pulse times from 0.22 to 26.29 s. The *Salmonella enterica* serotype Braenderup H9812 was used as the size marker. To determine the location of the *bla*_ND__M__–__1_ gene, DNA was transferred to a positively charged nylon membrane (Roche) and then hybridized with digoxigenin-labeled *bla*_NDM–__1_. Conjugation experiments were carried out in LB broth cultures. Azide-resistant *Escherichia coli* strain J53 [F(-) met pro Azi(r)] (recipient) and strain CBG15936 (donor) were mixed at a 1:3 ratio and then incubated at 37°C (Yi et al., 2012). After 18 h, transconjugants were selected for 12 h on MacConkey agar plates supplemented with meropenem (4 μg/ml) and sodium azide (150 μg/ml). Horizontal transferability of drug resistance was confirmed with antimicrobial susceptibility testing. Transconjugant-carrying plasmids were subsequently confirmed by PCR amplification and PFGE.

### Susceptibility Testing

The minimum inhibitory concentrations (MICs) of amikacin, aztreonam, nitrofurantoin, ciprofloxacin, piperacillin, gentamicin, cefepime, ceftriaxone, ceftazidime, cefotetan, cefazolin, tobramycin, imipenem, and levofloxacin were determined with the Vitek 2 Compact System (Bobenchik et al., 2015). *E. coli* reference strain ATCC25922 was used as quality control. The MIC value of meropenem was determined using an E-test. The results were interpreted according to Clinical and Laboratory Standards Institute [CLSI] (2018) guidelines.

### Whole-Genome Sequencing and Comparative Genome Analysis

Genomic DNA was extracted by using the High Pure PCR Template Preparation Kit (Qiagen, Inc., Valencia, CA, United States). Whole-genome sequencing was subsequently performed by using Illumina HiSeq according to the 350-bp paired-end protocol available from Novogene Company (Beijing, China) and MinION in our lab. The genome was assembled *de novo* by using a Unicycler (Li et al., 2010). RAST 2.0 was used to annotate the genome sequences obtained (Aziz et al., 2008). ResFinder v3.2 was used to identify acquired antibiotic resistance genes (Jia et al., 2016). Plasmid replicon type was analyzed by using PlasmidFinder (Carattoli et al., 2014). IS sequences were analyzed by using ISfinder (Siguier et al., 2006). Sequences of pNDM1-CBG and three similar plasmids were compared by using BLAST and Easyfig software (Sullivan et al., 2011). Whole-genome sequences of CBG15936 were used to determine multilocus sequence typing (MLST) based on detection of seven housekeeping genes (*arcA*, *aspC*, *clpX*, *dnaG*, *fadD*, *lysP*, and *mdh*) in pubMLST^1^ (Larsen et al., 2012). Twenty-nine sequences of the *hsp60* gene of eight *Enterobacter* clusters were retrieved from NCBI and used for phylogenetic analysis to identify the species of the strain.

### Nucleotide Sequence Accession Numbers

The entire sequence of strain, CBG15936, as well as plasmids, pTEM-CBG and pNDM1-CBG, have been deposited in GenBank under accession numbers CP046116, CP046117, and CP046118, respectively.

## Results

### Microbiological and Genetic Features of *E. cloacae* ssp. *dissolvens* CBG15936

**FIGURE 1:**
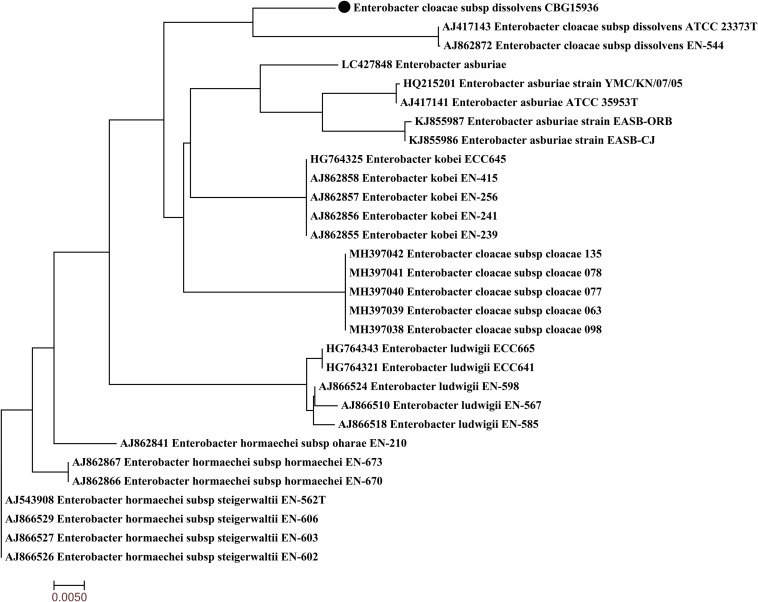
Phylogenetic tree of *Enterobacter cloacae* ssp. *dissolvens* strain CBG15936 with other 29 available *E. cloacae* complex *hsp60* genes from GenBank. Strain CBG15936 is marked with the black dot.

**FIGURE 2:**
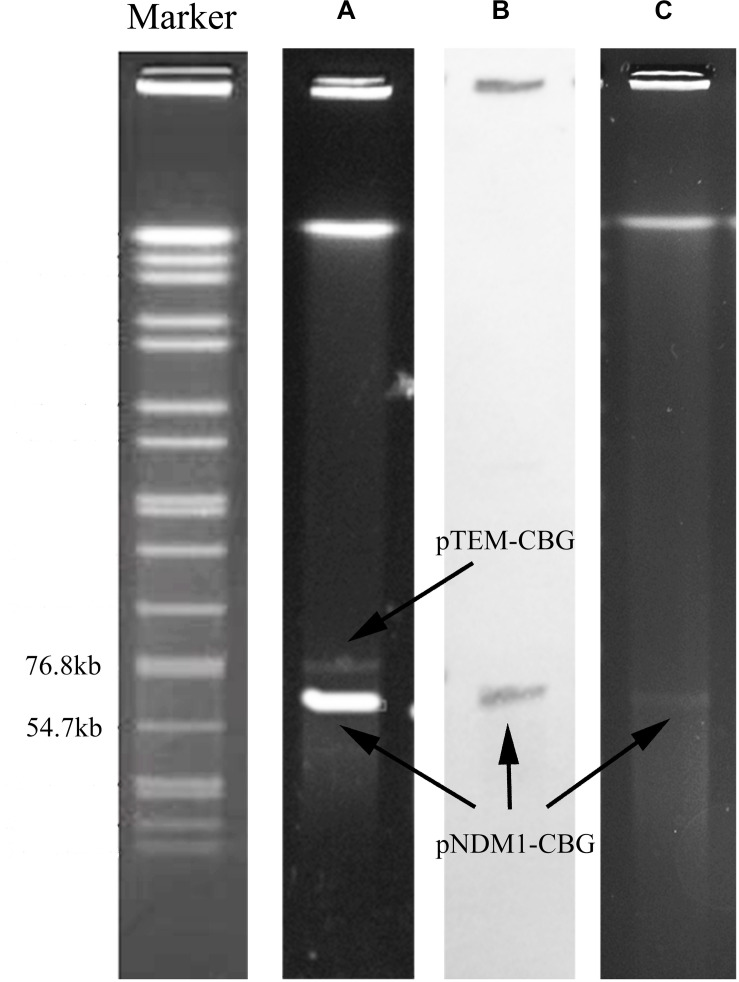
S1-PFGE pattern and Southern blotting for strain CBG15936 and *E. coli* J53 transconjugants. Lanes: Marker, *Salmonella* serotype Braenderup strain H9812 as a reference size standard. **(A)** PFGE result for S1-digested plasmid DNA of strain CBG15936. **(B)** Southern blot hybridization with probes specific to *bla*_NDM–__1_; **(C)** PFGE result for S1-digested plasmid DNA of strain *E. coli* J53 transconjugants.

Strain CBG15936 was identified as *E. cloacae* using Vitek and 16S rRNA and further classified as *E. cloacae* ssp. *dissolvens* based on *hsp60* genotyping (Figure 1). Susceptibility testing showed that *E. cloacae* ssp. *dissolvens* strain CBG15936 exhibits resistance to amikacin, aztreonam, piperacillin, cefepime, ceftriaxone, ceftazidime, cefotetan, cefazolin, and imipenem (Table 1). The E-test showed that strain CBG15936 (MIC ≥ 16) and transconjugants (MIC > 32) both exhibit resistance to meropenem. The strain was found positive for the *bla*_NDM–__1_ gene by PCR. S1-PFGE showed that this strain contains two different plasmids ∼60 and 75 kb in length (Figure 2). Southern blotting indicated that the *bla*_NDM–__1_ gene is present in the ∼60-kb plasmid. S1-PFGE and conjugation assays revealed that transfer of the plasmid carrying the *bla*_NDM–__1_, *qnrS1*, and *dfrA14* genes from strain CBG15936 to *E. coli* J53 occurs at a frequency of 1.68 × 10^–3^ events per donor cell (Figure 2). And the transconjugants were observed to acquire resistance to piperacillin, cefepime, ceftriaxone, ceftazidime, cefotetan, cefazolin, and imipenem (Table 1). According to MLST, strain CBG15936 is Sequence Type 932. Unicycler assembly results showed that this strain contains two plasmids with lengths of 75,044 and 62,663 bp. The ∼75-kb plasmid was designated pTEM-CBG, and it contains four drug resistance genes: *fosA3* (conferring fosfomycin resistance), *rmtB* (conferring aminoglycosides resistance), *bla*_CTX–M–__65_ (conferring cephalosporin resistance), and *bla*_TEM–__1__*B*_ (conferring penicillin resistance). The ∼62-kb plasmid was designated pNDM1-CBG, and it contains three resistance genes: *qnrS1* (conferring quinolone resistance), *dfrA14* (conferring trimethoprim resistance), and *bla*_NDM–__1_ (conferring carbapenem resistance).

**TABLE 1:** Antibiotic susceptibilities of CBG15936, transconjugants, and recipients.

Antibiotic	MIC (μg/ml)		Recipients (*E. coli* J53)
	CBG15936	Transconjugants (*E. coli* J53)	
Amikacin	≥64	≤2	≤2
Aztreonam	32	≤1	≤1
Nitrofurantoin	32	≤16	≤16
Ciprofloxacin	≤4	≤1	≤0.25
Piperacillin	≥128	≥128	≤4
Gentamicin	16	≤1	≤1
Cefepime	≥64	≥64	≤4
Ceftriaxone	≥64	≥64	≤4
Ceftazidime	≥64	≥64	≤1
Cefotetan	≥64	≥64	≤1
Cefazolin	≥64	≥64	≤1
Tobramycin	16	≤1	≤1
Imipenem	≥16	≥16	≤1
Levofloxacin	≤8	≤1	≤0.25

### Genetic Features of the *bla*_NDM–__1_-Carrying Plasmid

**FIGURE 3:**
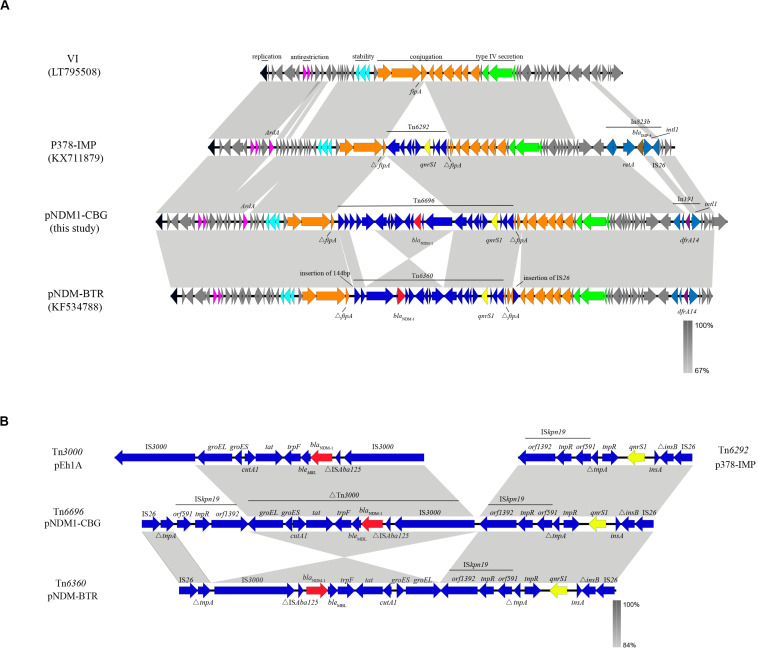
A comparative schematic diagram of **(A)** entire plasmids including VI (39,744 bp), p379-IMP (51,207 bp), pNDM1-CBG (62,663 bp), and pNDM-BTR (59,400 bp) and **(B)** transposons including Tn*6292* in p37-IMP, Tn*6696* in pNDM1-CBG, Tn*6360* in pNDM-BTR, and Tn*3000*. Open reading frames are indicated with arrows. Homology regions among different plasmids are denoted with light gray coloring. Gene backbones are shown in dark gray. Black, carmine, cyan, orange, and green arrows represent genes association with replication, antirestriction, stability, conjugation, and the type IV secretion system, respectively. Accessory modules are shown in blue; gene backbones of integron are shown in royal blue; *bla*_NDM–__1_ is shown in red; *qnrS1* is shown in yellow; *bla*_*IMP–*__4_ is shown in brown; *dfrA14* is shown in purple.

Plasmid pNDM1-CBG belongs to IncN1, has an average GC content of 52.34%, and has 91 open reading frames. Plasmid pNDM1-CBG also shares 99.95% identity and 100% coverage with plasmid pNDM-BTR, carried by *E. coli* BTR in Beijing, which was identified in 2013 (Zhao et al., 2017). Meanwhile, pNDM1-CBG shares 100% identity and 83% coverage with plasmid p378-IMP, carried by *Pseudomonas aeruginosa* in Chongqing, which was identified in 2013 (Feng et al., 2016). Both plasmids are composed of a similar ∼40-kb backbone and a variable multidrug resistance region. The backbone is composed of replication (*repA*), antirestriction (*ardA* and *klcA*), stability (*stdB*), conjugation (*tra*), and type IV secretion system (*virB*) genes. Compared with pNDM-BTR, pNDM1-CBG had an inversed ΔTn*3000*, an insertion of IS*kpn19*, a deletion of 144 bp upstream of the MDR region, and deletion of IS*26* downstream of the MDR region. Compared with p378-IMP, pNDM1-CBG is missing *ardA* downstream of the antirestriction region, yet it is present before the stability region. Replacement of In*823b* (which contains *bla*_IMP–__4_ downstream of the type IV secretion region) with In*191* (which contains *dfrA14*) is observed in plasmid pNDM1-CBG (Figure 3A).

The MDR region in pNDM1-CBG is composed of a novel transposon designated Tn*6696*. The latter consists of ΔTn*3000*, Tn*6292*, IS*26*, IS*kpn19*, and Δ*tnpA*. The *bla*_NDM–__1_ gene is located in ΔTn*3000* between a truncated IS*Aba125* and the *ble* gene. Compared with Tn*3000*, IS*3000* is deleted in ΔTn*3000*, which is organized as IS*3000*-ΔIS*Aba125-bla_NDM–__1_-ble-trpF-tat-*Δ*cutA1-groES-groEL*. Tn*6292* was first identified in p378-IMP and organized as *orf1393-tnpR-orf591*-Δ*trpA-tnpR-qnrs1-insA-*Δ*insB-*IS*26*. Tn*6696* is a combination of Tn*6292* and a truncated Tn*3000* and is similar to Tn*6360*, which was first identified in pNDM-BTR. Meanwhile, an inversed ΔTn*3000* and deletion of *ISkpn19* characterize Tn*6360* (Figure 3B).

## Discussion

*E. cloacae* have a broad range of hosts and have been isolated from wastewater, patients, and soil microcosms (Osborn et al., 2000; Chen et al., 2014; Zhang et al., 2014). Here, we report an NDM-1-producing MDR *E. cloacae* ssp. *dissolvens* strain, CBG15936, recovered from China. Our previous study showed that the expression of *bla*_NDM__–__5_ differed in plasmids and that the transconjugants with one plasmid exhibited higher-level carbapenem resistance than those with two plasmids or larger plasmids (Yang et al., 2020). Resistance to meropenem of transconjugants and strain CBG15936 may be affected by different NDM expressions and numbers of plasmids. This strain contains a novel transposon, Tn*6696*, carrying *bla*_NDM–__1_ and located on the IncN1 plasmid pNDM1-CBG. Considering broad-host-range plasmids generally occurs at a variable frequency from 10^–3^ to 10^–6^ (Grohmann et al., 2003), pNDM1-CBG exhibited a relatively high transfer frequency. Our results support the potential for cross-species transmission to occur.

The novel transposon, Tn*6696*, identified in pNDM1-CBG contains an inverted Tn*3000* remnant and an intact Tn*6292*. The latter is a Tn3-family transposon with an IS*26* at the right end and has previously been found in a pP378-IMP plasmid obtained from *P. aeruginosa* recovered in China (Feng et al., 2016). Tn*3000* was first located in a pEh1A plasmid from *Enterobacter hormaechei* E0083033-1 and in pEc2A from *E. coli* E0083033-2 recovered from Brazil (Campos et al., 2015). Therefore, a reorganization event involving fusion of Tn*3000* and Tn*6292* may have created Tn*6696*. The NDM-1 gene in Tn*6696* is bracketed by two copies of the insertion sequence, IS*kpn19*. It is hypothesized that the resulting special structure has the potential to mobilize a drug resistance gene (Wu et al., 2016). However, Tn*6696* had an additional copy of IS*kpn19* and an inverted ΔTn*3000* compared with Tn*6360*, suggesting that the former may originate from the latter through transposition and inversion of the IS*kpn19*-ΔTn*3000* structure.

Plasmid pNDM1-CBG also shares high similarity, yet lower coverage, with plasmid VI from *E. coli*. The main difference between these plasmids is the quite variable MDR regions, which are all inserted in the *fipA* gene at the same position. In a previous study, *fipA* was shown to be interrupted into two fragments by MDRs in many different plasmids (Zhao et al., 2017; Yang et al., 2018). Thus, *fipA* may serve as a “hot spot” for integration of mobile genetic elements.

## Conclusion

Here, we report a new transposon, Tn*6696*, in the IncN1 plasmid, pNDM1-CBG, from a ST932 MDR *E. cloacae* ssp. *dissolvens* strain recovered in China. We describe the structures of both pNDM1-CBG and Tn*6696* in detail. Our work provides a new perspective regarding the potential for a novel horizontal transfer of NDM-1 via Tn*6696* to occur among IncN1 plasmids. The latter finding is of great significance for future studies of the dissemination of *bla*_NDM–__1_ in different species and its monitoring.

## Data Availability Statement

The datasets generated for this study can be found in the CP046116, CP046117, and CP046118.

## Author Contributions

PL, HM, and HS conceived and designed the experiments. QC, LL, YL, and KW performed the experiments. ZL, PHL, and LY analyzed the data. QC and PL wrote the manuscript. All authors read and approved the final manuscript.

## Conflict of Interest

The authors declare that the research was conducted in the absence of any commercial or financial relationships that could be construed as a potential conflict of interest.

## Footnotes

1 https://pubmlst.org/mlst/
